# From Discovery to Cure—Where Are We Now? Mortality Trends in Chronic Hepatitis C: An Analysis of CDC WONDER Database (1999–2023)

**DOI:** 10.3390/v18050576

**Published:** 2026-05-20

**Authors:** Ashraf Ullah, Hina Wazir, Abdullah Sultany, Khalil Ur Rehman, Mohammad Ibrahim Sultani, Naeem Ahmed Khan, Saeed A. Khan, Mati Ullah Dad Ullah, Amlish Gondal

**Affiliations:** 1 Department of Internal Medicine, Guthrie Robert Packer Hospital, Sayre, PA 18840, USA; abdullahsultany142160@gmail.com (A.S.); amlish.gondal@guthrie.org (A.G.); 2 Jamaica Hospital Medical Center, Richmond Hill, New York City, NY 11418, USA; wazirhina3@gmail.com; 3 Department of Internal Medicine, Carle Foundation Hospital, Urbana, IL 61801, USA; khalilurrehmantajik@gmail.com; 4 Faculty of Medicine, Ankara Yıldırım Beyazıt University, Ankara 06800, Türkiye; ibrahim.sultani220@gmail.com; 5 Department of Internal Medicine, NYC Health + Hospitals/Woodhull, Brooklyn, NY 11206, USA; drnak451@gmail.com; 6 Ghurki Trust Teaching Hospital, Lahore 54000, Pakistan; saeedahmedkhan800@gmail.com; 7 Department of Internal Medicine, The University of Oklahoma, Norman, OK 73019, USA; matiu694@gmail.com

**Keywords:** hepatitis C, mortality, direct-acting antivirals, health disparities, CDC WONDER

## Abstract

Background: Hepatitis C virus (HCV) remains a major cause of preventable liver-related mortality in the United States despite highly effective direct-acting antivirals (DAAs). Contemporary assessment of mortality trends and disparities is essential for elimination efforts. Methods: Using CDC WONDER multiple cause-of-death data (1999–2023), we identified HCV-related deaths using ICD-10 codes for acute and chronic HCV (B17.1, B18.2) and calculated age-adjusted mortality rates (AAMRs) per 100,000 (2000 US standard). Rates were stratified by sex, race/ethnicity, census region, and 2013 NCHS urban–rural classification. Joinpoint regression quantified temporal inflection points and annual percent changes (APCs). Results: Overall HCV-related AAMR increased from 1.8 (1999) to a peak of 5.0 (2014), then declined to 2.3 (2023), with a marked post-2014 decrease (APC −8.2%). Mortality was consistently higher in males than females (2023 rate ratio 2.57). In 2023, American Indian/Alaska Native individuals had the highest mortality (AAMR 8.7; rate ratio 3.48 vs. non-Hispanic White), followed by non-Hispanic Black individuals (AAMR 6.2; rate ratio 2.48). Mortality remained highest in the West and was higher in non-metropolitan than metropolitan counties (AAMR 2.8 vs. 2.3; rate ratio 1.22), with a slower post-2014 decline in non-metropolitan areas. Conclusions: Our findings indicate that while the DAA era has been associated with a substantial reduction in HCV-related mortality at the national level, this progress has not been uniform across all populations. Persistent excess mortality among Native American and non-Hispanic Black individuals may reflect inequities in the HCV care cascade, including screening, confirmatory testing, linkage to specialty care, insurance-related restrictions, and the high cost of antiviral therapy. These results highlight the need for policies and public health strategies that improve equitable and affordable access to curative HCV treatment.

## 1. Introduction

Hepatitis C virus (HCV) infection remains a major public health problem and a leading contributor to cirrhosis, hepatocellular carcinoma, and liver-related mortality. In the United States, chronic HCV infection has historically been concentrated among persons born during 1945–1965, reflecting transmission through prior healthcare exposures and injection drug use, with clinical consequences emerging decades after acquisition as progressive fibrosis leads to end-stage liver disease. From the late 1990s through the mid-2010s, HCV-related mortality rose steadily and, by 2013, deaths associated with HCV exceeded the combined mortality from many other nationally notifiable infectious diseases, emphasizing the magnitude of this epidemic [1,2,3,4,5,6,7,8].

The therapeutic landscape changed dramatically with direct-acting antivirals (DAAs). Interferon-free regimens introduced in 2013–2014 achieved sustained virologic response rates exceeding 95%, with improved tolerability and short treatment durations. Clinical and real-world evidence has demonstrated that cure reduces hepatic decompensation, hepatocellular carcinoma risk, transplant need, and mortality. Early national analyses suggested a shift in population-level mortality trends during the initial DAA scale-up period; however, contemporary assessments extending through recent years and comprehensively characterizing disparities across demographic and geographic strata remain limited [9,10,11,12,13,14,15,16,17].

Despite curative therapy, inequities persist across the HCV care continuum (screening, diagnosis, linkage, treatment initiation, and cure). American Indian/Alaska Native communities experience disproportionate HCV burden driven by structural determinants, limited access to specialty care, and geographic barriers, while non-Hispanic Black individuals face inequities related to delayed diagnosis, advanced fibrosis at presentation, and healthcare system barriers. Rural communities similarly experience reduced access to HCV treatment due to specialist maldistribution, travel distance, insurance limitations, and resource constraints. Concurrently, the opioid epidemic has shifted transmission patterns and increased acute HCV infections among younger people who inject drugs, particularly in rural and non-urban settings, raising concern for future morbidity and mortality without effective prevention and treatment [18,19,20,21,22,23,24,25,26,27].

To inform elimination efforts, updated national surveillance of mortality trends and inequities is needed, particularly in the post-2014 era and through the COVID-19 period. Using CDC WONDER multiple cause-of-death data from 1999 to 2023, we aimed to (1) characterize long-term temporal trends in HCV-related mortality and identify inflection points; (2) assess trends by sex, race/ethnicity, region, and urban–rural classification; and (3) quantify persistent disparities to support targeted strategies for HCV elimination by 2030 [28,29,30].

## 2. Materials and Methods

### 2.1. Data Source

Mortality data were obtained from the Centers for Disease Control and Prevention Wide-ranging Online Data for Epidemiologic Research (CDC WONDER) multiple cause-of-death database for the period 1999–2023 The database contains death certificate information from all 50 US states and the District of Columbia and is compiled by the National Center for Health Statistics. Population denominators provided within CDC WONDER were used for rate calculations [31,32,33]. Available online: https://wonder.cdc.gov/mcd.html. Accessed on 2 December 2025.

### 2.2. Case Definition

HCV-related deaths were identified using International Classification of Diseases, Tenth Revision (ICD-10) codes B17.1 (acute hepatitis C) and B18.2 (chronic hepatitis C). We used a multiple cause-of-death approach, including deaths in which HCV was listed as either the underlying cause or a contributing cause. This approach improves sensitivity for capturing HCV-associated mortality, as deaths from cirrhosis, hepatocellular carcinoma, or hepatic failure may list these conditions as the underlying cause with HCV recorded elsewhere on the death certificate [34,35].

### 2.3. Inclusion and Exclusion Criteria

We included all deaths among U.S. residents from 1999 to 2023 in which hepatitis C virus infection was listed anywhere on the death certificate as an underlying or contributing cause of death, using ICD-10 codes B17.1 for acute hepatitis C and B18.2 for chronic hepatitis C. Deaths were included regardless of sex, race/ethnicity, census region, or urban–rural classification. Deaths without hepatitis C listed on the death certificate were excluded. Because CDC WONDER provides aggregate death certificate data and does not allow individual-level clinical confirmation of coinfections, deaths were not separately stratified by HIV or HBV coinfection status. Therefore, HIV and HBV coinfection were not included as separate subgroup variables in this analysis. This limitation has been added to the manuscript, and future studies using linked individual-level clinical datasets should evaluate the impact of HIV and HBV coinfection on HCV-related mortality trends.

### 2.4. Variables and Stratification

Mortality rates were stratified by:

- Sex (male, female);
- Race/ethnicity (non-Hispanic White, non-Hispanic Black, Hispanic, American Indian/Alaska Native, Asian/Pacific Islander);
- Age group (25–34, 35–44, 45–54, 55–64, 65–74, ≥75 years);
- Census region (Northeast, Midwest, South, West);
- Urbanization level using the 2013 National Center for Health Statistics urban–rural classification (metropolitan vs. non-metropolitan) [36].

### 2.5. Age-Adjusted Mortality Rates

Age-adjusted mortality rates (AAMRs) per 100,000 population were calculated using the direct method with the 2000 US standard population. Rates were calculated overall and for each stratum [37].

### 2.6. Trend Analysis

Temporal trends were evaluated using Joinpoint regression (Joinpoint Regression Program, version 5.0.2; National Cancer Institute). Models were fit on log-transformed AAMRs with a maximum of three joinpoints. Annual percent changes (APCs) and 95% confidence intervals (CIs) were calculated for each segment. Statistical significance was determined using Monte Carlo permutation testing with α = 0.05 [38,39].

### 2.7. Disparity Measures

Rate ratios (RRs) with 95% CIs were calculated to compare mortality across demographic and geographic groups, using non-Hispanic White individuals, females, and metropolitan counties as reference categories.

### 2.8. Ethical Considerations

This study used publicly available, de-identified aggregate data and was not considered human subjects research; institutional review board approval was not required.

## 3. Results

### 3.1. Overall Mortality Trends

**Figure 1:**
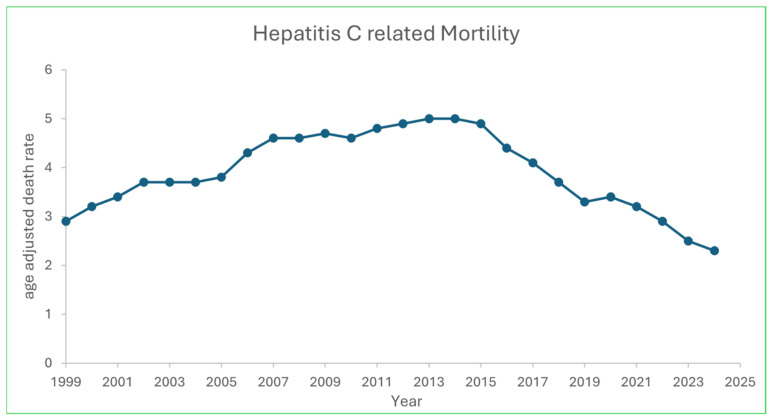
Age-Adjusted Mortality Rates for Hepatitis C–Related Deaths in the United States, 1999–2024.

Between 1999 and 2023, 487,326 HCV-related deaths were identified in the United States. The overall AAMR increased from 1.8 per 100,000 in 1999 to a peak of 5.0 per 100,000 in 2014, followed by a decline to 2.3 per 100,000 in 2023 (Figure 1). Joinpoint analysis identified 2014 as a significant inflection point (*p* < 0.001). From 1999 to 2014, mortality increased (APC +6.8%; 95% CI 6.4–7.2), whereas from 2014 to 2023 mortality declined (APC −8.2%; 95% CI −8.9 to −7.5). The absolute number of HCV-related deaths peaked at 21,432 in 2014 and decreased to 12,847 in 2023.

### 3.2. Sex-Specific Trends

Mortality was consistently higher among males than females. In 2023, the male AAMR was 3.6 per 100,000 compared with 1.4 per 100,000 among females (RR 2.57; 95% CI 2.48–2.66). Both sexes exhibited similar temporal patterns, with increases through 2014 followed by significant declines. The post-2014 APC was −8.5% (95% CI −9.3 to −7.7) in males and −7.6% (95% CI −8.5 to −6.7) in females.

### 3.3. Racial and Ethnic Disparities

**Figure 2:**
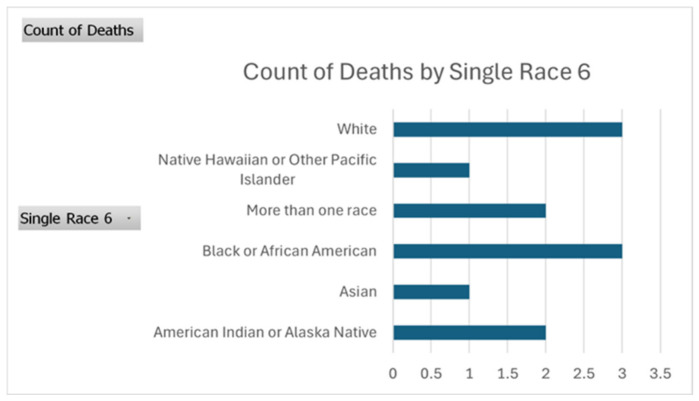
Distribution of Hepatitis C–Related Death Counts by Race/Ethnicity, United States, 2023.

Marked racial and ethnic disparities were observed. In 2023, American Indian/Alaska Native individuals had the highest mortality (AAMR 8.7 per 100,000; RR 3.48 vs. non-Hispanic White), followed by non-Hispanic Black individuals (AAMR 6.2; RR 2.48), Hispanic individuals (AAMR 4.1; RR 1.64), and Asian/Pacific Islander individuals (AAMR 1.8). All groups demonstrated declining mortality after 2014; however, the rate of decline was slower among non-Hispanic Black and American Indian/Alaska Native populations than among non-Hispanic White populations, resulting in persistent relative disparities (Figure 2).

### 3.4. Age Distribution

Mortality was highest among individuals aged 55–64 and 65–74 years. The 55–64 age group had a peak AAMR of 18.7 per 100,000 in 2014, declining to 8.2 per 100,000 in 2023. Approximately 78.6% of deaths occurred among individuals born between 1945 and 1965. Younger age groups had substantially lower mortality rates but showed modest increases in recent years.

### 3.5. Regional Variation

Geographic differences were observed across census regions. In 2023, the West had the highest mortality (AAMR 3.2 per 100,000), followed by the South (2.6), Midwest (2.1), and Northeast (1.8). All regions experienced post-2014 declines, with the most rapid reduction in the Northeast (APC −10.1%).

### 3.6. Urban–Rural Disparities

Non-metropolitan counties had higher mortality than metropolitan counties throughout the study period. In 2023, the AAMR was 2.8 per 100,000 in non-metropolitan areas versus 2.3 per 100,000 in metropolitan areas (RR 1.22; 95% CI 1.18–1.26). The decline after 2014 was slower in non-metropolitan areas (APC −7.4%) compared with metropolitan areas (APC −8.5%).

### 3.7. State-Level Variation

**Figure 3:**
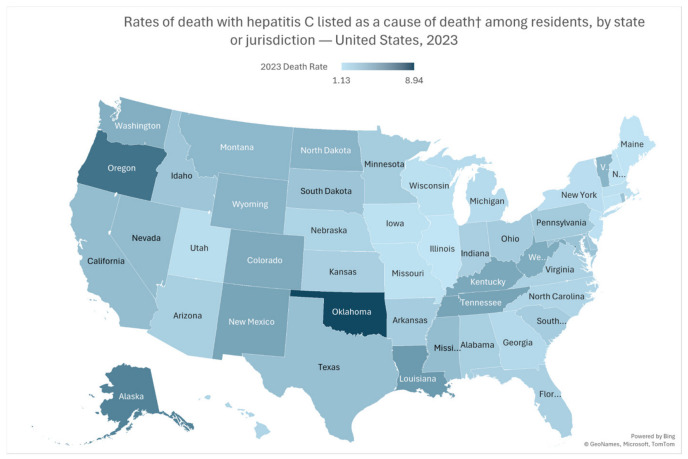
State-Level Variation in Hepatitis C–Related Mortality Rates, United States, 2023.

State-level analysis showed substantial geographic heterogeneity in HCV-related mortality in 2023. Oklahoma had the highest death rate (8.94 per 100,000), followed by the District of Columbia (7.56), Oregon (6.76), and Alaska (6.02), indicating a marked concentration of mortality burden in selected jurisdictions. Several states in the West and South ranked among the highest, including Louisiana, New Mexico, Colorado, Washington, and Nevada, while Appalachian and rural-burden states such as Kentucky and West Virginia also remained prominently affected. In contrast, the lowest death rates were observed in Illinois (1.13), New Hampshire (1.18), Maine (1.19), Connecticut (1.24), and New Jersey (1.31), with other large states such as New York (1.46) and Massachusetts (1.37) also showing relatively low mortality (Figure 3).

## 4. Discussion

In this national analysis of HCV-related mortality from 1999 to 2023, we observed a clear transition from rising mortality through 2014 to substantial declines thereafter. Joinpoint regression identified 2014 as an inflection point, with mortality shifting from a sustained annual increase to a marked post-2014 decrease [15]. These trends are consistent with population-level impact of DAA availability and scale-up. However, improvements were not evenly distributed: American Indian/Alaska Native and non-Hispanic Black populations experienced persistently higher mortality than non-Hispanic White populations, and non-metropolitan counties continued to have higher mortality and slower declines than metropolitan.

The magnitude and timing of the post-2014 decline are biologically and clinically plausible, given that DAA-mediated cure reduces hepatic inflammation, slows or reverses fibrosis progression, and lowers the risk of decompensation and hepatocellular carcinoma. At the population level, these benefits accumulate as treatment uptake expands and more individuals achieve cure before reaching irreversible end-stage disease. Nevertheless, mortality in 2023 remained above the 1999 baseline, suggesting incomplete reach of treatment and ongoing gaps in the care continuum. This persistent burden likely reflects undiagnosed infection, delayed linkage to care, residual risk among persons with advanced fibrosis or cirrhosis even after cure, and competing contributors such as substance use and comorbid metabolic disease [13,14,15,16,40,41].

The most striking inequities were observed among American Indian/Alaska Native populations, who had the highest mortality throughout the contemporary period. These disparities are likely multifactorial, including higher incidence related to structural determinants, differential access to harm reduction and addiction treatment services, geographic isolation, and healthcare under-resourcing. Importantly, misclassification of AI/AN race on death certificates may underestimate the true magnitude of disparity, meaning observed differences may represent a conservative estimate. Successful examples from tribal health systems—such as integrated screening and treatment programs and tele-mentoring/telehealth models that expand local prescribing capacity—indicate that high cure rates are achievable when access barriers are addressed [20,42,43,44].

Non-Hispanic Black populations also experienced substantially elevated mortality and slower relative improvements compared with non-Hispanic White populations, resulting in persistent or potentially widening relative disparities. Potential contributors include lower treatment uptake in earlier DAA years due to insurance restrictions, delayed diagnosis, differential access to specialty care, and structural inequities within healthcare delivery. These findings underscore that curative therapy alone does not guarantee equitable outcomes; policies and programs must address barriers at each step of the HCV care continuum through culturally responsive outreach, navigation, and removal of access restrictions [19,22].

We observed consistent urban–rural mortality differences, with non-metropolitan counties demonstrating higher mortality and a slower post-2014 decline than metropolitan counties. Rural communities face unique challenges, including limited specialist availability, transportation barriers, fewer harm reduction programs, and constraints in local health systems. The maldistribution of hepatology and infectious disease specialists particularly affects rural regions, where primary care clinicians may lack resources or training to manage HCV, despite guideline support for simplified treatment pathways in appropriate patients. Scalable models such as Project ECHO, telehealth consultation, and primary care–based treatment protocols can meaningfully improve access by enabling local treatment and reducing reliance on tertiary referral centers [24,44,45].

Regional differences—with the West exhibiting the highest mortality—likely reflect a combination of demographic composition (including higher proportions of AI/AN populations in some states), heterogeneity in injection drug use patterns, and variation in insurance coverage and policy implementation. State-level variation suggests that local structural factors, including Medicaid expansion decisions and DAA access restrictions, may influence the pace of mortality reduction [25,46].

Mortality was concentrated in older adults, consistent with the legacy cohort of chronic infections acquired decades earlier. However, increasing HCV incidence among younger adults in the context of the opioid epidemic represents a looming future burden if prevention, harm reduction, and treatment access are not strengthened. Achieving elimination will require dual focus: continued efforts to identify and treat the legacy cohort as well as integrated strategies for people who inject drugs, including linkage to addiction treatment, syringe service programs, and on-site or low-barrier DAA delivery. Evidence supports high cure rates in people who inject drugs, and treatment should not be withheld solely due to ongoing substance use [3,26,27,47].

The post-2014 mortality decline is encouraging and suggests real progress toward elimination targets; however, persistent disparities indicate that elimination will not be achieved equitably without targeted strategies. Priorities include: (1) universal and repeated screening where indicated; (2) rapid linkage to care with simplified treatment pathways; (3) removal of insurance and administrative barriers to DAA access; (4) expansion of treatment capacity in primary care and rural settings through telehealth and training; (5) culturally tailored programs and sustained investment in tribal health systems; and (6) integration of HCV services into addiction treatment, harm reduction, and correctional settings [30,41,46,48,49,50].

## 5. Limitations

This analysis has limitations inherent to death certificate data. HCV may be underreported as a contributing cause, potentially underestimating mortality; however, our use of multiple cause-of-death data improves capture relative to underlying-cause-only approaches. Ecologic temporal associations cannot prove causality, though the observed inflection aligns closely with DAA scale-up. Misclassification of race/ethnicity—particularly for AI/AN populations—may bias estimates downward. CDC WONDER lacks individual-level clinical data (e.g., fibrosis stage, treatment history, genotype, comorbidities), preventing mechanistic inference. Finally, mortality trends reflect both treatment and broader healthcare disruptions (including the COVID-19 period), which warrant further evaluation using complementary datasets [25,31,34,49] another limitation is that CDC WONDER provides aggregate death certificate data and does not include detailed individual-level clinical information. Therefore, we were unable to stratify HCV-related mortality by HIV or HBV coinfection status. HIV and HBV coinfection are clinically important because they may accelerate liver disease progression and contribute to worse outcomes among patients with HCV. Future studies using linked clinical datasets should further evaluate the impact of HIV and HBV coinfection on HCV-related mortality trends.

## 6. Conclusions

HCV-related mortality in the United States increased through 2014 and declined substantially thereafter, consistent with population-level benefits of DAAs. Nonetheless, marked racial/ethnic and geographic inequities persist, particularly among American Indian/Alaska Native populations and rural communities. Closing these gaps through policy reform and targeted delivery strategies is essential to achieve HCV elimination goals by 2030 [15,29,30].

## Data Availability

No new data were created or analyzed in this study.

## Abbreviations

AAMR, age-adjusted mortality rate; AI/AN, American Indian/Alaska Native; APC, annual percent change; CDC WONDER, Centers for Disease Control and Prevention Wide-ranging Online Data for Epidemiologic Research; CI, confidence interval; DAA, direct-acting antiviral; HBV, hepatitis B virus; HCC, hepatocellular carcinoma; HCV, hepatitis C virus; HIV, human immunodeficiency virus; ICD-10, International Classification of Diseases, Tenth Revision; NCHS, National Center for Health Statistics; NHB, non-Hispanic Black; NHW, non-Hispanic White; RR, rate ratio; SVR, sustained virologic response.
